# Elbow flexion contractures in neonatal brachial plexus palsy: A one-year comparison of dynamic orthosis and serial casting

**DOI:** 10.1177/02692155221121011

**Published:** 2022-08-24

**Authors:** L.S. Op de Coul, S. Bleeker, J.H. de Groot, R.G.H.H. Nelissen, D. Steenbeek

**Affiliations:** 1 Department of Rehabilitation, 4501 Leiden University Medical Center, Leiden, Netherlands; 2 Department of Rehabilitation, Hand & Pols Centrum, Den Haag, Netherlands; 3 Department of Orthopedics, 4501 Leiden University Medical Center, Leiden, Netherlands

**Keywords:** Child rehabilitation, neurological disability, goal attainment scaling, orthoses, range of movement

## Abstract

**Objective:**

Elbow flexion contractures are common complications of neonatal brachial plexus palsy, but evidence on how to treat these contractures is weak. This study compared the treatment of elbow flexion contractures using a dynamic orthosis or serial circular casting.

**Methods:**

A randomized controlled trial was conducted with one-year follow-up. Children with an elbow flexion contracture of ≥30° were treated with either a night-worn dynamic orthosis for one year or serial casting for four weeks followed by night splinting. For pragmatic reasons, some participants were included in an open part of this study, this group was also analyzed separately. Degree of contracture and goal attainment scaling was evaluated at baseline and after 8, 20 and 54 weeks.

**Results:**

55 patients were analyzed in this trial, 32 of whom were randomized to treatment. At one-year follow-up of the randomized group, both dynamic splinting (median −8.5°, interquartile range [IQR] −13.5, −5) and serial casting (median −11.0°, IQR −16, −5) resulted in significant reduction of contracture (*P* < 0*.*001). The reduction was significantly greater with serial casting in the first 20 weeks, but not at one-year follow-up (*P* = 0.683). In the entire cohort, the individual functional goals had been reached in 24 out of 32 cases (80%) of dynamic splinting and 18 out of 23 cases (82%) of serial casting, respectively.

**Conclusion:**

The dynamic night orthosis is comparable to serial casting for treating elbow flexion contractures in children with brachial plexus birth injury. We recommend selecting one of these treatment modalities in close consultation with parents and patients.

## Introduction

Neonatal brachial plexus palsy (NBPP) is a flaccid paresis of the upper extremity caused by trauma to the brachial plexus during delivery, resulting in damage to several or all of the C5–8 and T1 spinal roots. The incidence of NBPP varies between 1.5 and 4.6 per 1000 live births.^1,2^ The natural course of the condition is known to vary greatly and depends on the extent and severity of the nerve lesions. Up to 34% of NBPP patients will have residual muscle weakness and will thus not make a full neurological recovery.^3^

A common complication of NBPP is an elbow flexion contracture, which limits the maximum extension angle of the elbow. These contractures develop on average at the age of five years. The median prevalence of elbow flexion contractures in children with NBPP is 48%, and 21%–36% of them have a contracture of more than 30°.^4,5^ Despite all efforts from parents and therapists by passively stretching the elbow, the development of a more serious contracture is sometimes inevitable. For decades, the etiology of elbow flexion contractures in children with NBPP remained elusive. However, a recent review illustrated that multiple factors influence their occurrence, with reduced growth of the affected flexor muscles caused by denervation most likely playing the greatest role.^4^

Since most daily living activities are performed in 30°–150° of elbow flexion,^6,7^ the inability to extend the elbow to more than 30° of flexion results in functional limitations during daily activities, such as reaching out in front of and above the body. Treatment is therefore considered to be important and can be nonsurgical or surgical.^6–9^ As for the latter, this is mainly an option for a persistent elbow flexion contracture of more than 50°–60°. Since the quality of evidence on the effectiveness of treatment options for elbow flexion contractures in children with NBPP is weak, nonsurgical interventions are the treatment of the first choice.^10^

One of these nonsurgical options is serial casting of the elbow. This intervention has proved to achieve significant improvement in passive range of motion (PROM) for elbow extension.^5,11,12^ However, the interpretation of the results is limited by their overall weak validity, due to small study samples, heterogeneity and poor study methodology.^10^ Possible complications of this treatment are an inability to flex the elbow due to serial casting, radial head dislocation and recurrence of the elbow flexion contracture.^12^ An advantage of treatment with serial casting is high adherence to the therapy, since the cast cannot be removed by the participants or their parents.

Another nonsurgical option is the use of a dynamic orthosis. There have been no studies regarding the use of this particular orthosis in children with NBPP and severe elbow flexion contractures. However, this orthosis has been studied in adults with posttraumatic elbow stiffness and showed good results.^13^ The pathophysiology of posttraumatic elbow stiffness is likely to be different from that of elbow flexion contractures in children with NBPP, since it includes not only soft-tissue contractures but also heterotopic ossification, extra-articular and intra-articular malunions, nonunions, and loss of articular cartilage.^14^ The effect of an orthosis might therefore be different as well. Moreover, the long-term effects are likely to be different in children of different ages, since growth may also play a part. The dynamic orthosis applies a prolonged, mildly stretching force to the ligaments, joint capsule, elbow flexor muscles and possibly other tissues like cartilage. One could imagine that this is more comfortable than the continuous force from the rigid casts. Complications that were observed during serial casting are less likely to occur during treatment with a dynamic orthosis, since the elbow can be moved as usual during the period the orthosis is not used (daytime) and since the dynamic spring hinge allows for active elbow flexion. If the dynamic orthosis should prove to be as efficient as serial casting for treating elbow flexion contractures in children with NBPP, then presumed complication reduction and continuity of activity could make the dynamic orthosis preferable to serial casting. Our study, therefore, compared the treatment of elbow flexion contractures in children with NBPP using a dynamic orthosis or serial circular casting.

## Methods

A single-blind randomized controlled trial with a one-year follow-up was conducted between 2013 and 2017, including 60 consecutive patients who visited the nerve injury outpatient clinic. The core task of the nerve injury outpatient clinic is to diagnose and follow up children with NBPP from birth to the age of 18 years, addressing functional limitations in a multidisciplinary team setting, consisting of three neurosurgeons, two orthopedic surgeons, two (pediatric) rehabilitation physicians (physiatrists), a physical therapist, and an occupational therapist. Included in this study were children with either an elbow flexion contracture ≥30° or with rapid progression of the elbow flexion contracture, defined as a progression of ≥10° in six months, both occurring in spite of intensive stretching exercises and physical therapy. Exclusion criteria were previous casting or splinting of the elbow, proven radial head dislocation on X-ray and inability of the child and/or parents to follow the instructions regarding the treatment.

Patients were block-randomized, based on four defined age categories. If patients or parents did not want to participate in the randomization, for example, because of presumed discomfort of the therapy or inability to participate in sports, they were allowed to opt for a specific type of treatment. The follow-up appointments and outcome parameters of this so-called “open inclusion group” were according to the RCT- study protocol below. This allowed us to include the open inclusion group in our analyses as well. The study protocol was registered and approved by the medical ethics committee of the Leiden University Medical Center and registered by the national central committee on research involving human subjects (NL43722.058.13). Patients and parents were informed about the possible discomforts and complications of the treatment they would receive. They were provided information by their rehabilitation physician; it consisted of both oral explanation and flyers. Since both treatment options were part of regular care in our outpatient clinic, there were no preferences on the part of the practitioners, ensuring that objective information was provided to the patients and parents. Written informed consent was obtained prior to the study.

**Figure 1.**
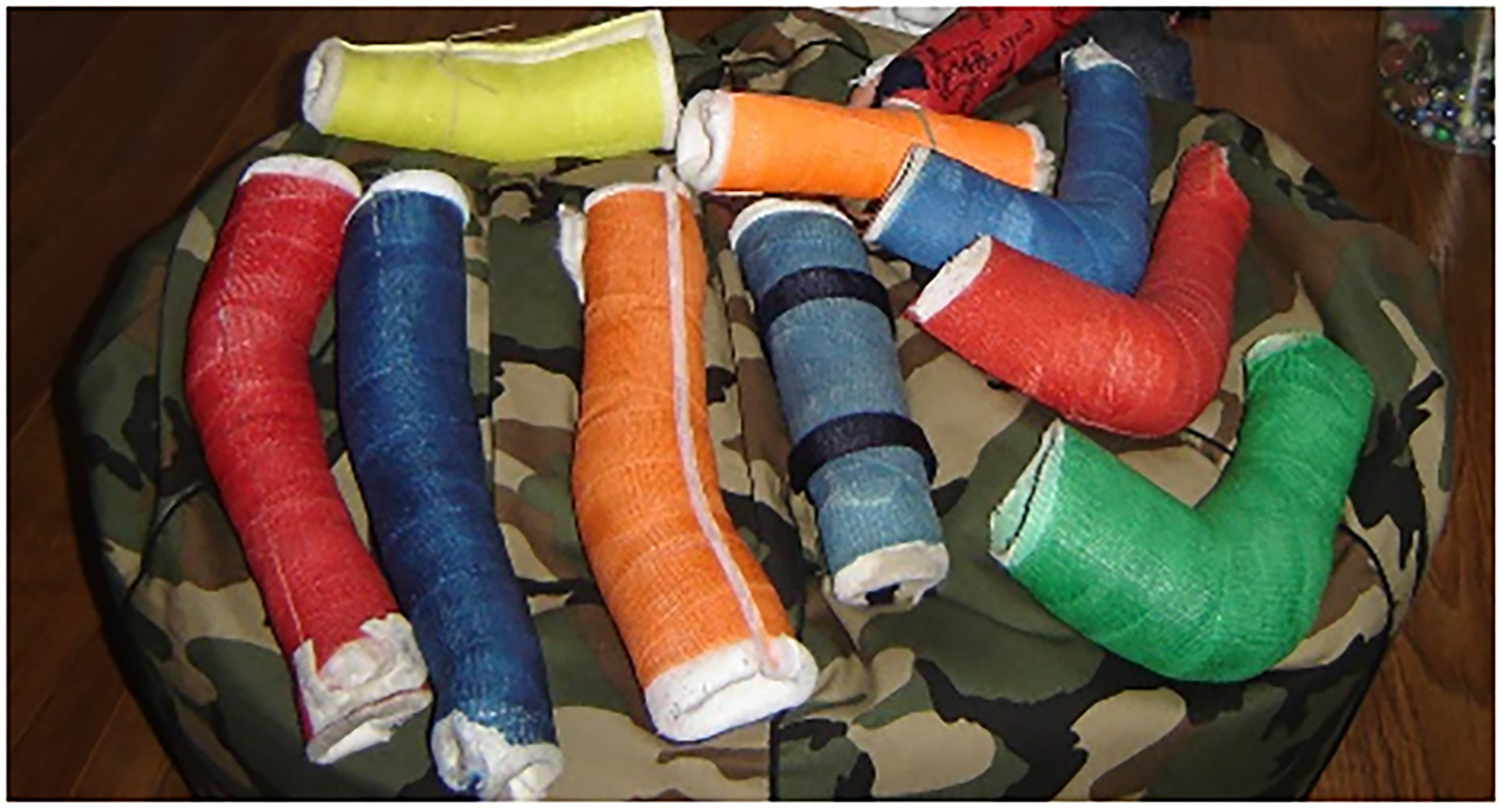
Serial casting.

**Figure 2.**
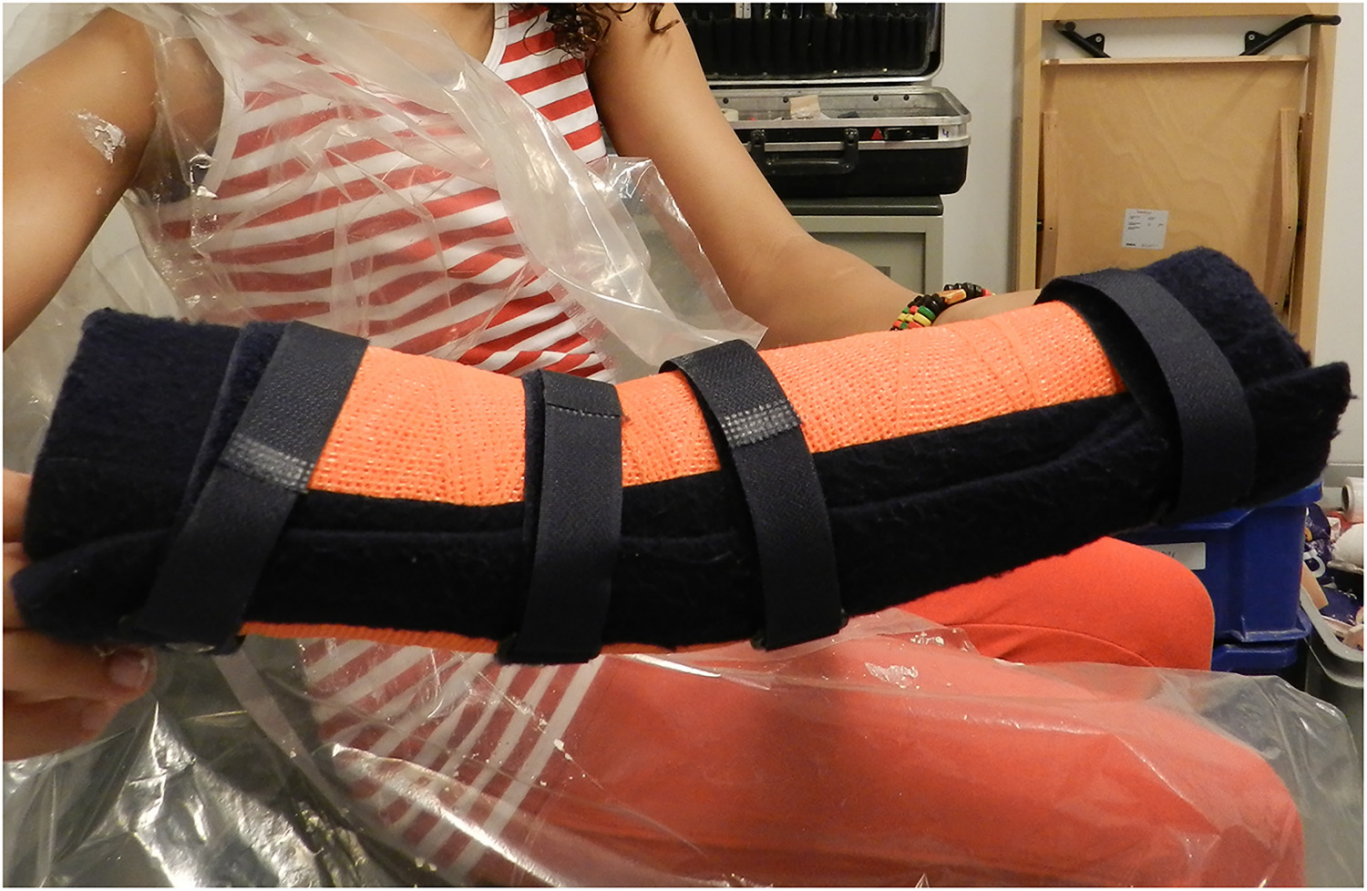
Removable night splint.

During the study, physical therapy or rehabilitation programs were continued without change of therapy strategy, irrespective of the allocated group. Serial casting (Figure 1) was performed by our experienced orthopedic casting technicians over a period of four weeks with a weekly cast change. During this cast change, the elbow was passively and actively moved over the full range of motion, to prevent complications as reported by Duijnisveld et al.^12^ The new cast was constructed based on the prevailing PROM of the elbow in extension. The forearm was in a neutral to mild supinating position, offering the most comfortable position. In order to prevent a recurrence, serial casting was followed by night splinting with a removable cast in maximum elbow extension, fixated with four Velcro straps, for the remaining weeks up to the final follow-up evaluation after 54 weeks of treatment (Figure 2). The night splint was only replaced in case of wear, for example, caused by dirt. Any other causes for replacement of the splint were registered.

**Figure 3.**
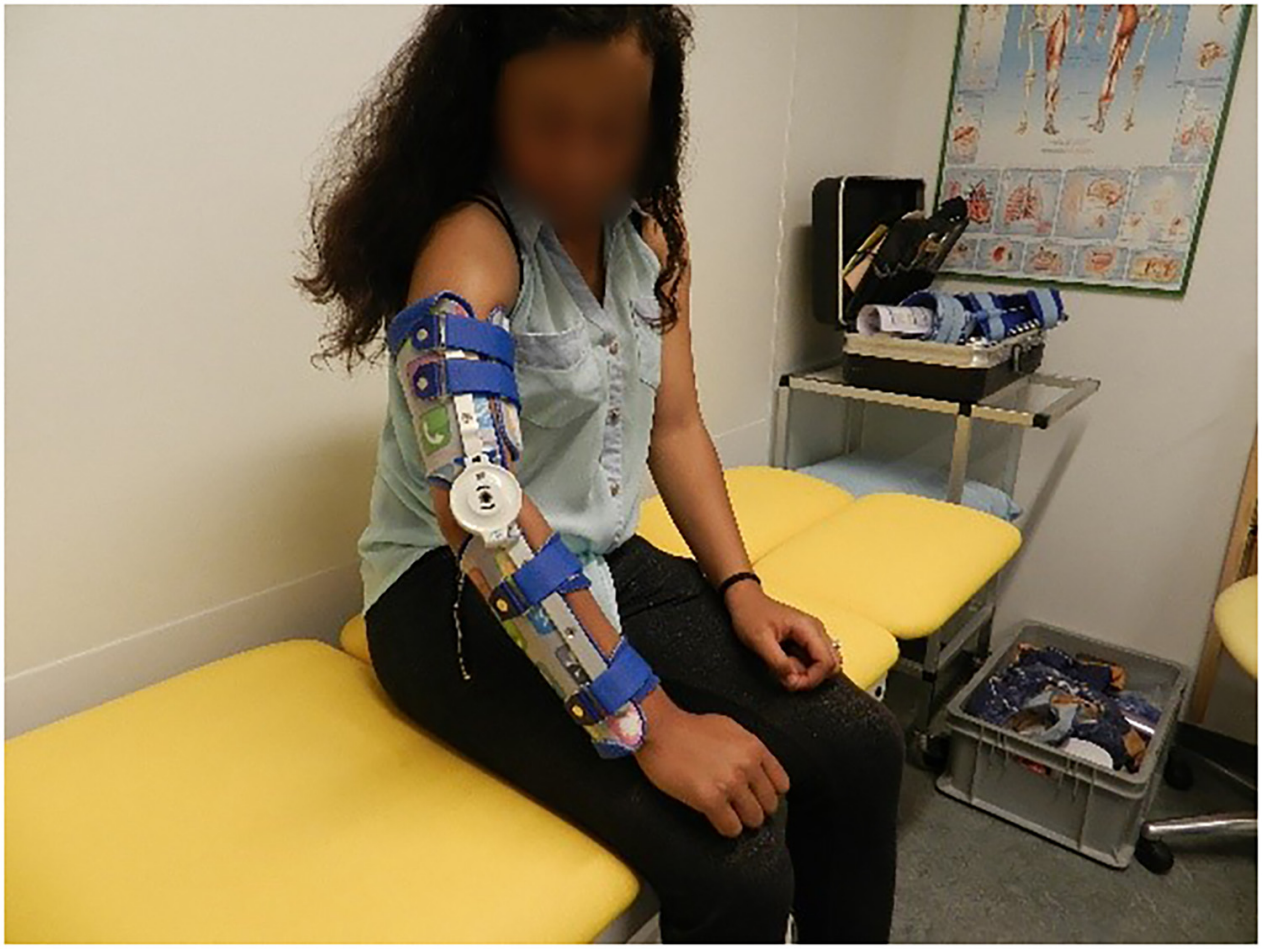
Dynamic orthosis.

The dynamic orthosis (Figure 3) was constructed by a certified prosthetist and orthotist, who was associated with our hospital. Our standard orthosis consisted of polypropylene dorsal scales on the upper arm and forearm. These scales were connected by a dynamic spring hinge, providing an adjustable extension force. A separate scale was placed ventrally on the upper arm, as proximally as possible, and was fixed with Velcro straps. This facilitated optimized comfortable distribution of three-point pressure. Comparable to the position of the forearm in the casts, the axial forearm rotation ranged from a neutral position to mild supination, whichever ensured the greatest comfort. The orthosis was prescribed to be worn every night until the final follow-up evaluation after 54 weeks of treatment. Parents were instructed to adjust the spring hinge to provide for a constant, mildly stretching force on the elbow joint. During the two follow-up appointments at 8 and 20 weeks, the hinge was checked and adjusted if necessary, and adherence to instructions was checked.

**Figure 4.**
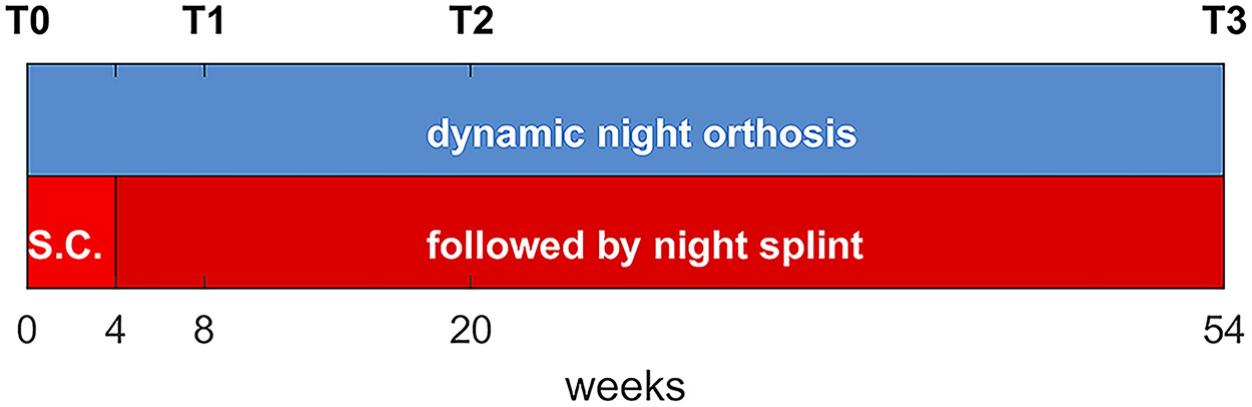
Timeline of treatment.

Clinical parameters such as sex, age, affected side, Mallet score, Narakas type and previous surgical treatments were recorded at inclusion. All patients were evaluated at baseline (T0) and after 8 (T1), 20 (T2), and 54 weeks (T3) of treatment (Figure 4).

**Figure 5.**
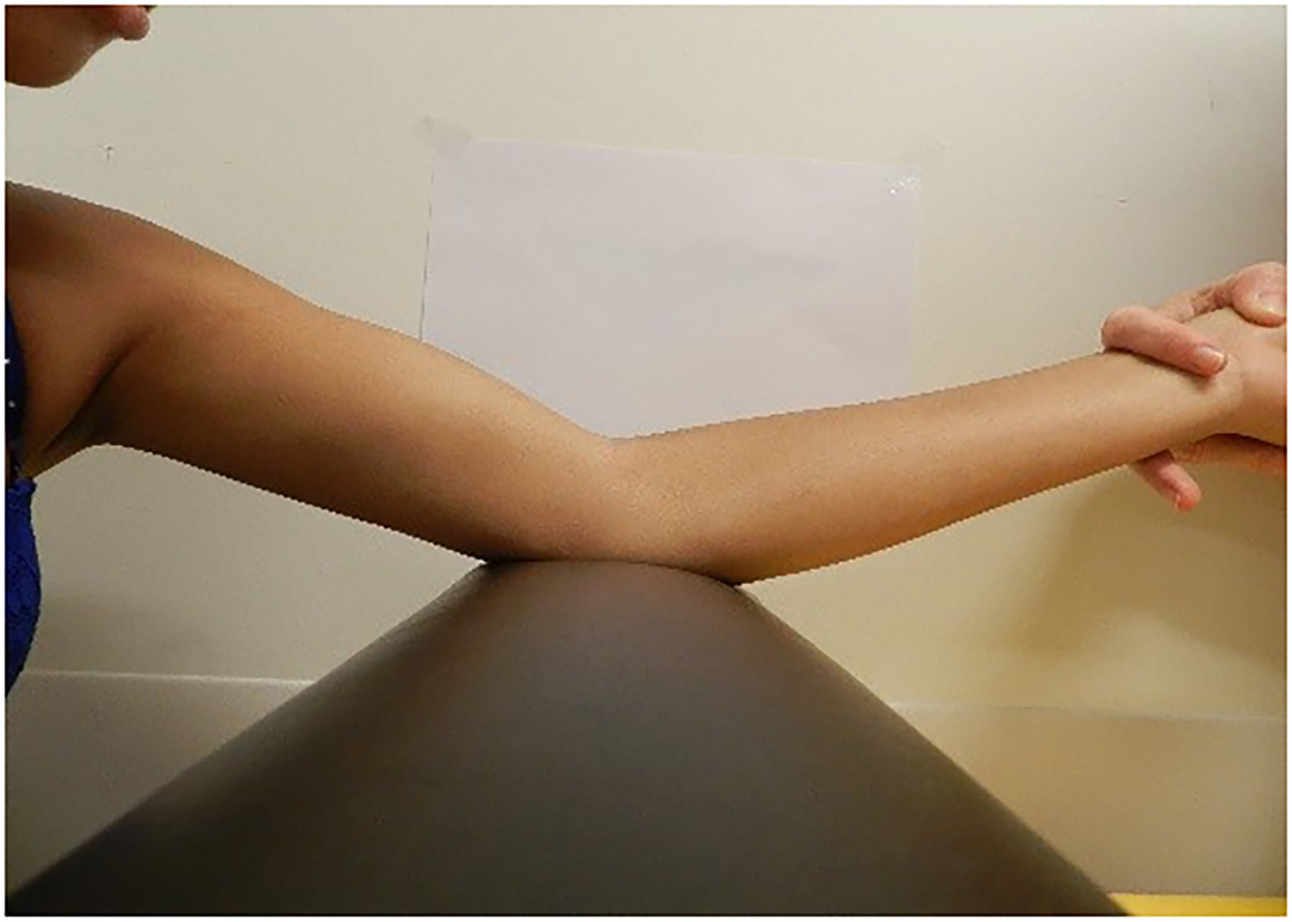
Photograph perpendicular to the plane of the passively stretched elbow.

The degree of elbow flexion contracture, the primary outcome of this study, is included at the body functions and structure level in the International Classification of Functioning, Disability and Health for Children and Youth (ICF-CY). At each appointment, a photograph was taken perpendicular to the plane of the flexed arm, while the elbow was passively extended by our experienced physiotherapist until the resistance of the elbow occurred (Figure 5). This measurement was performed in the maximum achievable supination of the forearm, as this provides for tension release of the biceps muscle and hence for a reliable measurement with the goniometer. In random order, elbow angles were measured from these photographs, using a goniometer, always by the same, independent and blinded researcher (SB). The inter- and intrarater reliabilities (two raters, SB and JdG, blinded for treatment) were calculated for a randomized sample consisting of the first 95 measurements.

Outcomes from the activity domain of the ICF-CY were measured at every follow-up appointment with goal attainment scaling (GAS), using the adjusted 6-point version of the original GAS method.^15,16^ A topical and individually important activity was chosen, which, in the opinion of the team (pediatric rehabilitation physician, physiotherapist and occupational therapist), was closely related to the elbow flexion contracture. Subsequently, the participant's own rehabilitation physician constructed and scored GAS scales in consultation with the children and their parents. In this scale, a score of −2 represents a level equal to baseline, −1 represents less progress than expected, 0 represents the functional goal that was set, scores of +1 and +2 represent the achievement of more and much more, respectively, than was expected, and finally a score of −3 represents a deterioration of functioning.^15^ We analyzed raw GAS scores instead of applying the commonly used original T-sum formula.^17^

Other secondary outcomes included therapy comfort and self-reported adherence to the treatment, as reported at the one-year follow-up. We recorded the retrospective opinion of patients and parents; there were no interim reports. Therapy comfort was reported after one-year follow-up on a 10-point scale, in which zero equaled no comfort and 10 indicated that the therapy was very comfortable. Adherence to therapy was expressed as the percentage of time that the patient was adherent to the therapy, with 100% indicating complete adherence to the study protocol.

### Statistics

The primary outcome was a reduction of elbow flexion contractures over a period of 54 weeks. A power analysis indicated that a sample size of 55 patients would provide 80% power (α = 0.05, effect size = 0.15) to detect a clinically relevant difference of 10° between treatment options.

Statistical analysis was performed with IBM SPSS Statistics 25 and R Configuration Extension. Statistics were used to assess the possibility of analyzing the randomized patients and the patients included by open inclusion together. Descriptive statistics were used for baseline characteristics. The primary outcome, the reduction of elbow flexion contracture, was analyzed using generalized estimating equations, in order to correct for possible confounders such as randomization, age at inclusion, and sex. A comparison of GAS scores between treatment groups during the course of the study was analyzed with a continuation ratio model. Reported therapy comfort and self-adherence were compared between the two groups using a nonparametric test.

## Results

**Figure 6.**
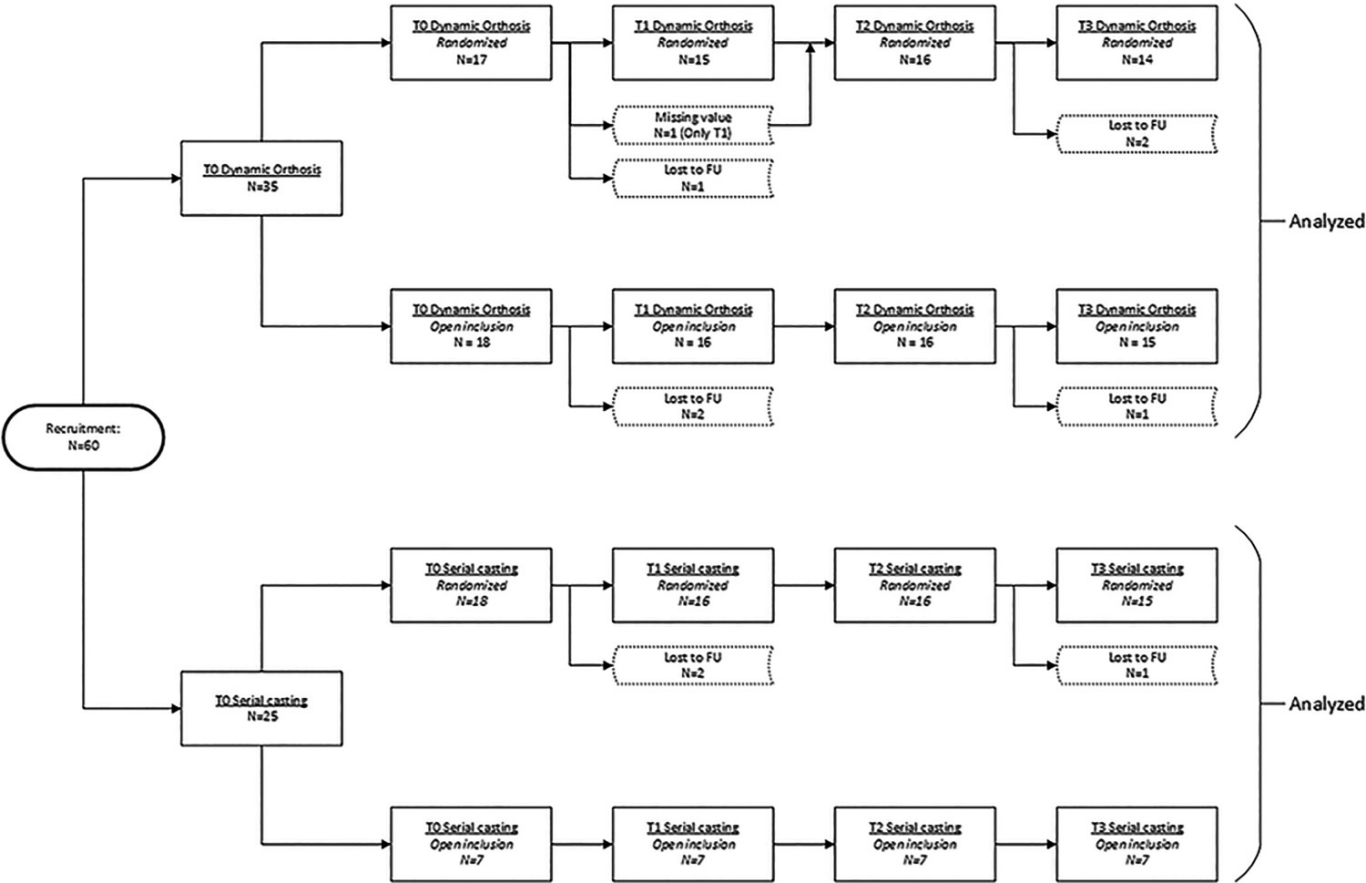
Patient flow diagram. Dotted lines indicate missing values.

Thirty-five patients were included for randomization and were treated with either the dynamic orthosis (*n* = 17) or serial circular casting (*n* = 18). As illustrated in Figure 6, the personal preference group consisted of an additional 25 patients (18 dynamic orthoses, 7 serial castings), bringing the total in the overall group to 60 patients. Preference for open inclusion in the dynamic orthosis cohort was based on presumed therapy comfort (*n* = 3), being able to participate in sports such as swimming or dancing (*n* = 6), and attending school without a cast (*n* = 7), whereas the choice for serial casting was based on adherence to therapy (*n* = 6) and presumed effect of treatment (*n* = 1). Five patients dropped out between baseline and first evaluation, their reasons varying from lack of motivation to coincidental medical problems or change of treatment modality. These patients were excluded from the analysis. Patients with no more than two missing values were included in the analysis. This meant that 32 randomized patients were included in the statistical analysis, whilst the overall group consisted of 55 patients.

Table 1 shows baseline patient characteristics for both treatment options. The primary outcome was analyzed in the randomized group (with additional analysis in the overall group). The secondary outcomes were calculated for the entire cohort, since these subjective measurements are very difficult to analyze in a small sample size.

**Table 1.** Patient characteristics at baseline.

**Treatment**	**Dynamic orthosis (** ***N* = 32)**		**Serial casting (** ***N* = 23)**	
	**Open inclusion** (***N* = 16)**	**Randomized (** ***N* = 16)**	**Open inclusion (** ***N* = 7)**	**Randomized (** ***N* = 16)**
**Sex (M/F)**	7/9	8/8	3/4	5/11
Age (years, months)*	11,4 (4.0)	9,7 (3.5)	10,5 (4.1)	11,0 (2.7)
** 2–4 years**	1	2	0	2
** 5–8 years**	3	2	1	2
** 9–11 years**	4	6	3	4
** 12–18 years**	7	6	3	8
**Affected side (R/L)**	11/5	7/9	3/4	8/8
**Narakas type**				
** C5**–**C6**	3	7	3	5
** C5**–**C7**	6	5	3	8
** C5**–**T1**	7	4	1	3
Elbow flexion contracture**	37.5	37.0	42.0	39.0
	(32.25,41.75)	(32,39.75)	(33,45)	(32.5,42)
Passive pronation**	70 (80,90)	90 (80,90)	80 (0,90)	90 (27.5,90)
Passive supination**	80 (55,90)	90 (72.5,90)	90 (80,90)	90 (82.5,90)
**Previous treatment**				
** Nonsurgical**	1	4	0	2
** Neurosurgery**	5	6	3	10
** Neurosurgery and orthopedic surgery**	8	5	4	4
** Neurosurgery and botulin toxin A**	1	0	0	0
** Orthopedic surgery**	1	1	0	0
Mallet score**				
** Shoulder abduction**	3 (3,4)	3.5 (3,4)	4 (4,4)	4 (3,4)
** Shoulder external rotation**	3 (1,4)	2 (1.25,3)	2 (1,4)	2 (1,3)
** Hand to head**	3 (2,3)	3.5 (3,4)	3 (3,4)	3 (3,4)
** Hand to back**	3 (2,4)	2 (2,4)	3 (2,4)	2 (2,4)
** Hand to mouth**	3 (3,4)	3 (3,4)	3 (3,4)	3 (3,4)

*Mean at baseline (SD).

**Median at baseline (IQR).

IQR: interquartile range.

### Elbow flexion contracture

Intrarater reliability and inter-rater reliability were calculated for measuring the degree of an elbow flexion contracture from a photograph. With an intrarater reliability of ICC = 0.992 and an inter-rater reliability of ICC = 0.948, the method was found to be excellent for use in this trial.

At the 54-week follow-up, the two groups in the randomized cohort had achieved reductions of elbow flexion contracture of −8.5° (median, dynamic orthosis, interquartile range [IQR] −13.5, −5) and −11° (median, serial casting, IQR −16, −5), respectively (*P* < 0.001). No difference was observed between the two cohorts at one-year follow-up. Table 2 presents the results of the randomized and the entire cohort for each interval. The analysis with generalized estimating equations was applied to the entire cohort, and after inclusion of possible confounders such as randomization, age at inclusion and sex, it confirmed the absence of a difference at one-year follow-up.

**Table 2.** Reduction of elbow flexion contracture over time.

Interval (weeks)	Dynamic orthosis (IQR)		Serial casting (IQR)		Significance	
	Randomized	Complete	Randomized	Complete	Randomized	Complete
0–8	7° (2,13)	9° (5,13)	13° (6,18)	13° (10,18)	*P* = 0.11	*P* = 0.01
8–20	8° (4,17)	8° (5,17)	13° (9,16)	15° (10,20)	*P* = 0.05	*P* = 0.03
20–54	9.0° (5,14)	9° (5,17)	11° (5,16)	13° (6,16)	*P* = 0.68	*P* = 0.55

IQR: interquartile range.

**Figure 7.**
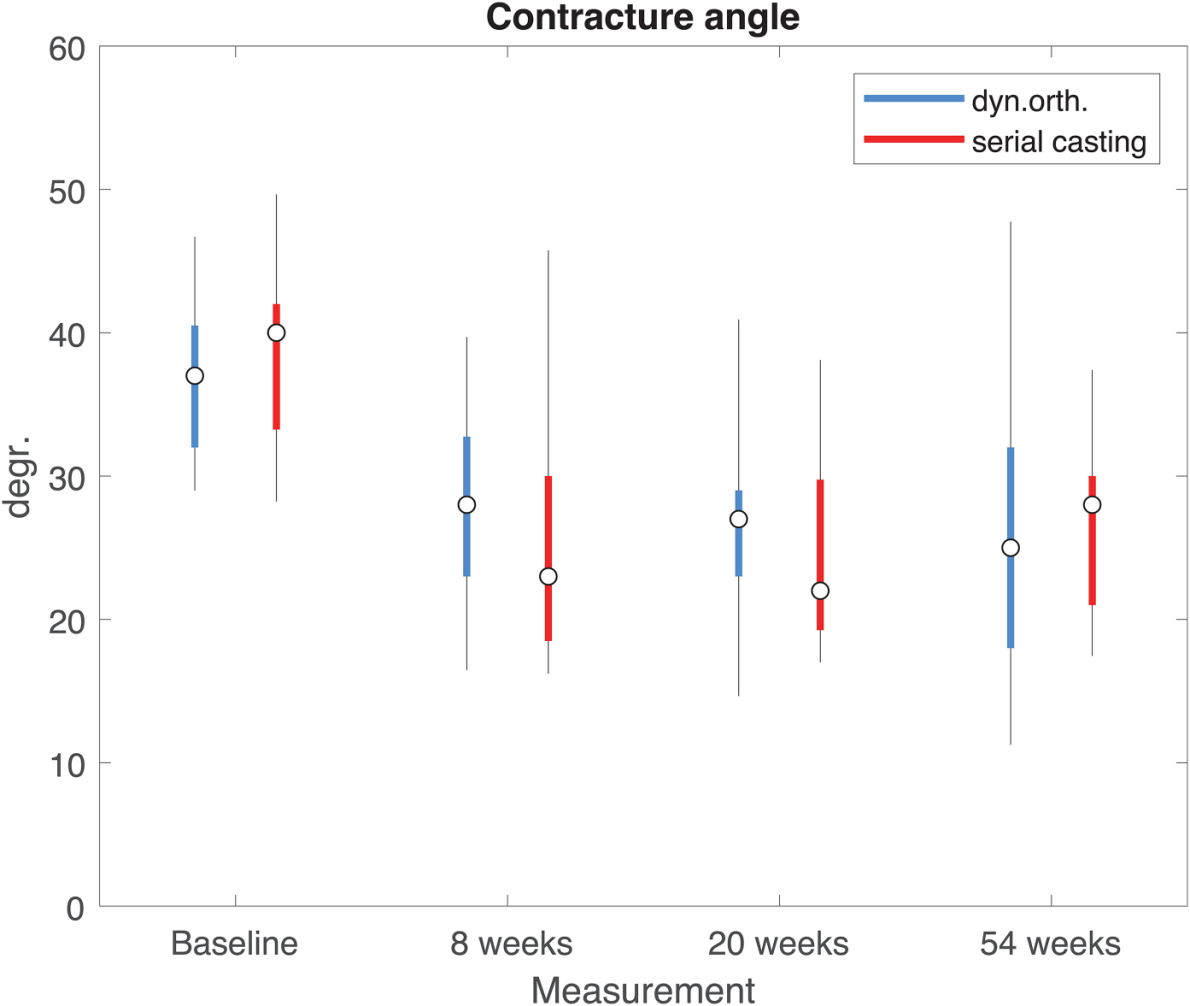
Boxplot of contracture over time for each treatment. Whiskers are based on the (7.5, 92.5) percentiles.

Figure 7 illustrates the reduction of elbow flexion contracture over time with serial casting and the dynamic orthosis for the overall group, that is, including both the randomized and personal preference groups. Both dynamic splinting (median −9.0°, IQR −17, −5) and serial casting (median −13.0°, IQR −16, −5.75) resulted in a significant reduction of elbow flexion contracture at the final evaluation (*P* < 0*.*001). Serial casting initially provided faster reduction of elbow flexion contracture, as was found at the T0–T1 interval (*P* = 0.011). Between 8 and 24 weeks, the contracture angle remained constant, and the difference between serial casting and dynamic splinting was preserved (*P* = 0.031). The difference between the treatment options disappeared between 25 and 54 weeks (*P* = 0.53).

### Goal attainment scaling

Table 3 presents an overview of the different categories of GAS scores in each treatment group, and their median GAS scores at each follow-up appointment. Categories were defined based on corresponding individual goals set by the patients and their parents. The remaining unique goals were included in the “other” category. The table shows improvements in the different categories of goals set.

**Table 3.** Overview of GAS values.**^A^**

Category (goal)	Dynamic orthosis/serial casting		Median GAS at T1		Median GAS at T2		Median GAS at T3	
	Rand.^C^	Comp.^D^	Rand.	Comp.	Rand.	Comp.	Rand.	Comp.
Riding a bike	4/3	7/5	−1	−1	−0.5^B^	0^B^	1**^B^**	1^B^
Support (push up)	2/2	7/4	−0.5	−1	1	−1	2^B^	1^B^
Ball skills	6/1	7/1	−1	−1	0	0	1	1
Swimming	1/0	2/0	0	−0.5	0	0	1	1
Dancing & performing martial arts	1/0	3/1	−1	−1.5	0	−0.5	0	−0.5
Esthetics	0/6	4/7	0.5	−1	0.5	0	1^B^	0^B^
Other	2/4	2/5	−1	−1	0.5	1	1	1

^A^ Baseline value −2, range −3 to 2.

^B^ 1 missing value.

^C^ Randomized cohort.

^D^ Complete cohort.

**Figure 8.**
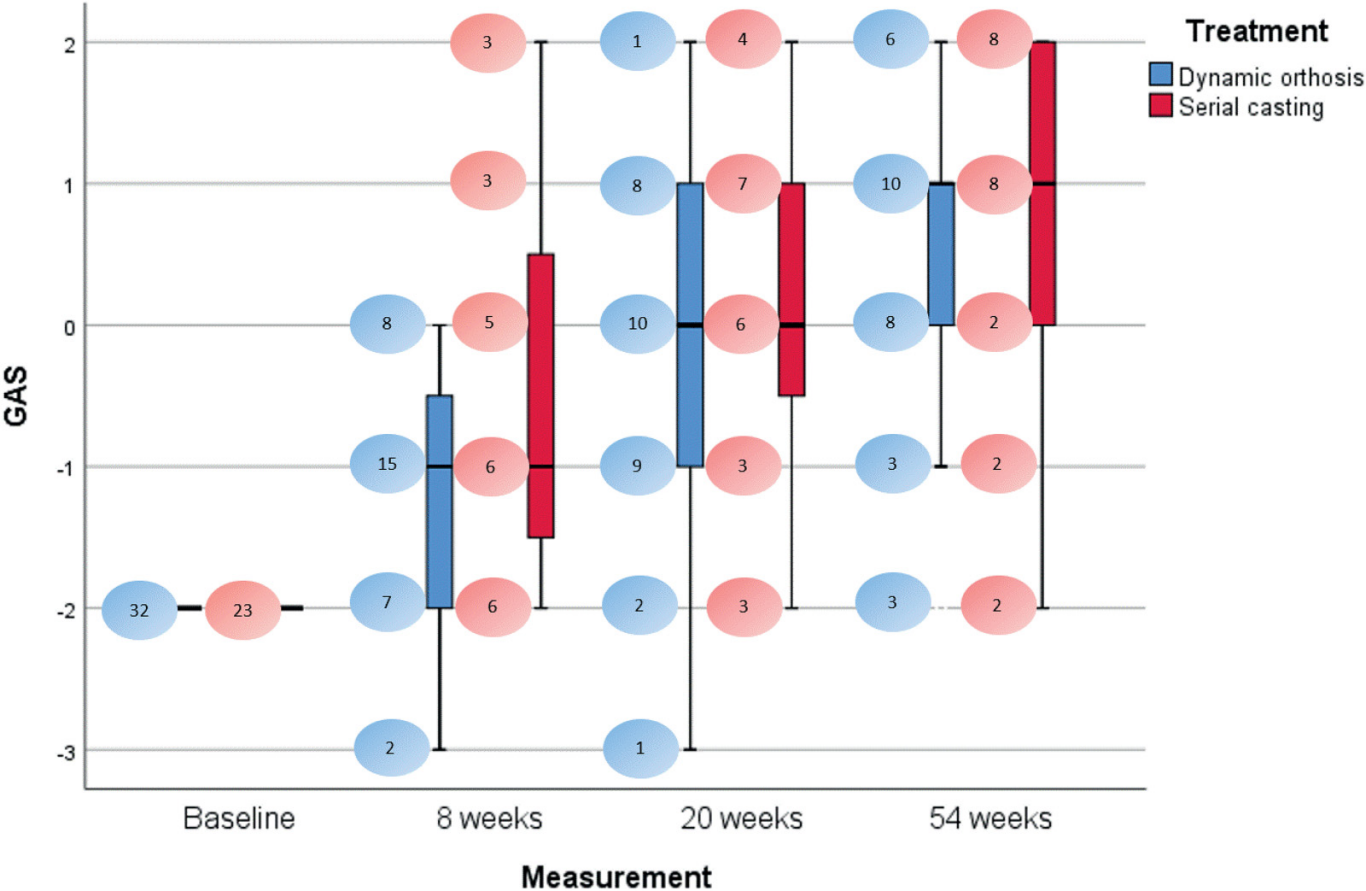
Boxplot of GAS outcomes over time for each treatment. Outliers have been removed (Q1-1.5*IQR and Q3 + 1.5*IQR); whiskers are based on the maximum and minimum values not being outliers. Bubbles represent numbers of cases for every GAS score per treatment and measurement. IQR: interquartile range.

The individual goals at the activity level were reached in 24 (out of 30) and 18 (out of 22) cases for the dynamic orthosis and serial casting groups, respectively. Figure 8 displays the distribution of GAS scores at each measurement for the entire cohort and for each treatment group. The shift from lower to higher GAS scores is clearly visible and comparable for both treatments. Median improvement in GAS between baseline and final evaluation was 3 points for both serial casting and dynamic orthosis (*P* < 0.001). Statistical analysis confirmed that the progress in GAS scores did not differ between the two treatment groups (*P* = 0.21). The same model showed that the reduction of elbow flexion contracture correlated with the change in GAS scores over time (*P* < 0.001).

### Therapy comfort, adherence to therapy and complications

The median score for the comfort of the dynamic orthosis in the total group was 8.00 (IQR 7.00, 8.25) (randomized cohort: 7.5, IQR 7.00, 8.25), compared to a median of 7.00 (IQR 5.00, 7.00) for serial casting followed by night splinting (and the same for the randomized cohort: 7.00, IQR 5.00, 7.00). The Wilcoxon Signed Rank test revealed a significant difference in comfort in favor of the dynamic orthosis in the entire cohort (*P* = 0.041), as well as in the randomized cohort (*P* = 0.043).

When analyzing the entire cohort, the median self-reported adherence to therapy using the dynamic orthosis was 90% (IQR 50, 97.5), which was comparable to the median adherence using the night splint in the serial casting cohort, namely 85% (IQR 70, 95, *P* *=* 0.860). The analysis of the randomized cohort showed the same adherence (*P* *=* 0.860). No complications were reported during the course of this study. No other reasons for replacement of the night splint (besides wear) were registered.

## Discussion

The objective of the current study was to compare two methods (dynamic night orthosis and serial circular casting followed by night splinting) for the treatment of an elbow flexion contracture of more than 30° in children with NBPP. Both treatment options showed a comparable reduction in the elbow flexion contracture one year after the start of treatment, with serial casting providing a faster initial decrease of flexion contracture within the first 20 weeks, compared to the dynamic orthosis. Night splinting after serial casting may prevent contracture relapse but did not lead to further reduction of contracture, due to the static nature of the splints. The dynamic orthosis can be regarded as a prolonged treatment, which could explain the persistent effect of dynamic splinting up to 54 weeks, in contrast to a slight relapse of contracture with night splinting in the serial casting cohort.

Improving overall functioning and, for that matter, changing the overall functioning of a child with NBPP, should be the ultimate aim of any intervention. Goal attainment scaling is one of the most responsive methods to measure change over time and differences between treatment strategies, as the most relevant goals are individually set and evaluated.^18^ GAS evaluation by one of the members of an experienced treatment team is considered to be a reliable method, despite the subjectivity of this outcome, as long as the evaluator has no conflicts of interest.^19^ If GAS records change over time, possible therapist bias has to be considered. If GAS does not record any change, actual change at the activity level is unlikely. When assessing our functional results as shown by GAS scores, we can conclude that most participants reached the functional goals they defined at baseline, regardless of the treatment they received. In the present study, we did not find relevant differences between the treatment groups at the activity level of the ICF-CY. Although a relationship was found between reduction of elbow flexion contractures and GAS scores, a limitation of measuring outcomes at the activity level is the possible use of compensation. It is likely that improvement over time in GAS scores is partially due to children learning how to compensate for their disability, which could lead to an overestimation of the treatment effect. For example, in the ball skills category, a participant's goal was set at dribbling a basketball, which improved because he started to move his entire trunk to provide a bouncing force on the basketball, rather than extending his elbow further.

Retrospectively self-reported adherence to treatment and treatment comfort at one-year follow-up did not differ between treatments in the randomized cohort. For the entire cohort, comfort was scored one point higher (median) on the 10-point scale for dynamic orthosis. Although this difference was statistically significant, we do not consider this one-point difference to be clinically relevant. Comfort was measured at the final evaluation only. Since serial casting was only applied in the first four weeks of treatment, followed by night splinting, this outcome may be biased in favor of serial casting. Measuring comfort at each appointment could have provided a more reliable outcome, since measuring during the casting period would presumably have led to more discomfort being reported in the serial casting cohort.

Since some parents and children showed a clear prior preference for one of the treatment modalities, we decided to allow treatment by choice, in order to include children who refused (or whose parents refused) randomization due to a strong preference for a particular treatment strategy. This treatment choice could have biased the results due to differences in, for instance, motivation and adherence to therapy. The absence of an effect of randomization as a significant factor in the generalized estimating equations analysis, supports our opinion that (in the shared decision-making about treatments) both options are well suited for treating elbow flexion contractures in children with NBPP.

There were limitations to our study design. First of all, for pragmatic reasons, our analysis included not only the randomized patients but also children treated with their or their parents’ preferred modality. This created selection bias, but it also reflects daily clinical practice. Most parents in this NBPP patient population are intensely involved with their child and its condition, while they are also very well aware of the sequelae of this severe injury. Secondly, both treatment options, except for the weeks of serial casting, depend on the adherence to therapy of children and their parents. We do not know whether our instructions were followed closely during the course of treatment, since this was only retrospectively reported. This again, however, corresponds to daily practice and is therefore representative of real-life treatment. Both therapy options rely on the alertness of patients and parents, orthopedic casting technicians and orthotists. For future research, we would recommend evaluating adherence to treatment at each follow-up appointment instead of the retrospective evaluation in the current study. Additionally, we recommend investigating the state of the dynamic orthosis and night splint, in order to objectify the actual use. In our opinion, the serial casting method is reproducible, and protocols for manufacturing the dynamic orthosis can be developed. In this way, both treatments can be standardized as much as possible.

No cost analysis was performed during this study. The costs of manufacturing a dynamic orthosis will differ between countries, as will the expenses covered by insurance companies. However, the costs of serial casting will also differ, as these are defined by factors such as travel time and plaster room costs. This could influence the availability of a particular treatment modality for patients.

In conclusion, the dynamic night orthosis is comparable to serial circular casting followed by night splinting for the treatment of elbow flexion contractures in children with NBPP. We recommend that specialists make a decision together with patients and their parents, based on treatment characteristics, individual factors, and costs. For future research, we recommend studying the effect of serial casting for rapid contracture reduction followed by treatment with a dynamic orthosis for prolonged treatment.

**Clinical messages**

- After one year, reduction of contractures in children with NBPP showed no difference between treatment with serial casting and a dynamic orthosis. Serial casting seems to initially provide greater reduction of these contractures.
- Serial casting and a dynamic orthosis led to similar functional goal attainment at the activity level of the International Classification of Functioning, Disability and Health for Children and Youth.
- We recommend choosing between the treatment modalities needs to be in a shared decision with patients and caregivers.

## Appendix

**Table A1.** Pronation and supination at baseline and after one year of treatment.^A^

Treatment	Pronation		Supination	
	Baseline	Final evaluation	Baseline	Final evaluation
Dynamic orthosis	90 (80,90)	90 (70,90)	90 (72.5,90)	90 (80,90)
Serial casting	90 (27.5,90)	85 (41.25,90)	90 (82.5,90)	90 (82.5,90)
^A^Median (IQR)				

IQR: interquartile range.

**Table A2.** Pronation and supination at baseline, related to Narakas classification.^A^

Narakas classification	Pronation	Supination
C5-C6	90 (80,90)	90 (80,90)
C5-C6-C7	80 (35,90)	90 (85,90)
C5-C6-C7-C8 ± Th1	90 (0,90)	90 (0,90)
^A^Median (IQR)		

IQR: interquartile range.
